# Aeration, Agitation and Cell Immobilization on Corncobs and Oak Wood Chips Effects on Balsamic-Styled Vinegar Production

**DOI:** 10.3390/foods8080303

**Published:** 2019-08-01

**Authors:** Ucrecia F. Hutchinson, Sivuyile Gqozo, Neil P. Jolly, Boredi S. Chidi, Heinrich W. Du Plessis, Maxwell Mewa-Ngongang, Seteno K. O. Ntwampe

**Affiliations:** 1 Post-Harvest and Agro-Processing Technologies, ARC Infruitec-Nietvoorbij (The Fruit, Vine and Wine Institute of the Agricultural Research Council), Private Bag X5026, Stellenbosch 7599, South Africa; 2 Bioresource Engineering Research Group (BioERG), Department of Biotechnology, Cape Peninsula University of Technology, P.O. Box 652, Cape Town 8000, South Africa; 3 Department of Chemical Engineering, Cape Peninsula University of Technology, P.O. Box 652, Cape Town 8000, South Africa

**Keywords:** aeration, adsorption, balsamic-styled vinegar, cell immobilization, corncobs, non-*Saccharomyces* yeast, oak wood chips

## Abstract

Optimum fermentor conditions are essential for desired microbial growth and activity in fermentations. In balsamic vinegar fermentation systems, the microorganisms used must endure several stressful conditions including high sugar concentration, low water activity, high osmotic pressure and high acetic acid concentration. Consequently, the present study was aimed at improving the performance of a microbial consortium of non-*Saccharomyces* yeast and acetic acid bacteria during balsamic-styled vinegar fermentation. Cell immobilization via adsorption on corncobs and oak wood chips in combination with aeration and agitation effects, have never been tested during balsamic-styled vinegar fermentation. Therefore, fermentations were initially conducted under static conditions without aeration with successive fermentations also being subjected to low (0.15 vvm min^−1^) and high (0.3 vvm min^−1^) aeration. The results showed improved acetification rates when cells were immobilized on corncobs under static conditions. Low aeration showed better acetification rates (1.45–1.56 g·L·day^−1^), while only free-floating cells were able to complete fermentations (1.2 g·L·day^−1^) under high aeration conditions. Overall, cells immobilized on corncobs showed higher acetification rates of 1.56 and 2.7 g·L·day^−1^ under low aeration and static fermentations, respectively. Oak wood chips were determined to be less efficient adsorbents due to their relatively smooth surface, while the rough surface and porosity of corncobs led to improved adsorption and, therefore, enhanced acetification rates.

## 1. Introduction

Similar to other vinegars, balsamic vinegar is a food flavoring agent or condiment, which contains acetic acid as its main ingredient [1]. Balsamic vinegar is characterized by its sour and sweet taste [2] and can be used as a salad dressing, and for soothing sore throats among other uses [3]. Balsamic-styled vinegar (BSV) is a term adopted from Traditional Balsamic vinegar (TBV) of Modena and Reggio-Emilia Italy. BSV is a new type of Balsamic vinegar which has potential for being produced in South Africa using Chenin blanc wine grapes, since they are the most cultivated grape cultivar in South Africa [4].

Cell immobilization generally refers to a technique, which is performed to prevent cells from being freely suspended in the fermentation medium [5,6,7]. Cell immobilization improves biomass growth, increases cell stability, protects cells from the toxic environment, and provides a reusability option [8]. Most fermentations generally employ the free-floating cell (FFC) method, which is effective in less stressful fermentation systems. However, during balsamic vinegar fermentation, there are multiple factors that have antagonistic effects on the microorganisms involved in the fermentation. For instance, the fermentation medium (cooked grape must) used for Balsamic-styled vinegar has a very high sugar content, low water activity and high osmotic pressure [9,10,11]. Furthermore, the high concentrations of acetic acid during fermentations can also have a negative impact on the microbial consortium used [12,13]. These aforementioned environmental stresses can cause the microbial consortia to enter the viable but non-culturable state, resulting in reduced microbial activity [14,15]. Consequently, to counteract these effects, cell immobilization is an ideal approach, which can minimize the impact of stressful conditions on the microorganisms used. 

Various methods are used to perform cell immobilization. These include entrapment in a gel matrix, immobilization on a solid surface and mechanical containment behind a barrier [16]; however, to date, numerous studies focus on cell immobilization via gel entrapment or immobilization on a solid surface (adsorption) [5]. In Balsamic-styled vinegar fermentation, cell immobilization has previously been studied using the gel entrapment technique [17], which is generally an expensive technique. However, cell immobilization by adsorption in combination with aeration and agitation effects has never been tested for balsamic vinegar production. Therefore, in this current study, cell immobilization on corncobs and oak wood chips was investigated using the adsorption technique, which is commonly classified as a simple and often cost-effective method depending on the support material used [8]. 

Corncobs are an inexpensive and abundant agricultural by-product obtained from the corn-milling process. They are currently used as animal feed, with 80% of their dry matter being comprised of cellulose and hemicellulose [18,19,20,21,22]. These aforementioned characteristics impart unique attributes that brand corncobs as an ideal material for cell immobilization. On the other hand, oak wood chips are abundantly used as an alternative to wooden barrel aging in the wine industry for economic reasons. They are known to improve the sensorial qualities of the final product due to the flavors they impart to wines [23,24,25]. For this reason, the current study selected oak wood chips, based on both their cell immobilization properties and potential impartation of organoleptic characteristics to the BSV. Nevertheless, oak wood chips are usually imported to South Africa and they cost more than corncobs. Consequently, this study investigated readily available inert materials which can be obtained at varying cost for cell immobilization, in order to allow producers to make an informed decision in achieving the desired BSV quality. Furthermore, the study explored the capabilities of the selected materials for cell affinity to the adsorbents selected and fermenter performance in both aerated and agitated cultures systems.

## 2. Material and Methods

### 2.1. Preparation of Fermentation Medium

The study commenced with the boiling of Chenin blanc grape must (22 °Brix) using a double jacketed steam pot (S.W.18, Aluminium Plant and Vessel Co. Ltd., London, UK) until a sugar concentration of 30 °Brix was obtained. The grape must was aliquoted into 3 L Erlenmeyer flasks, covered with cotton wool stoppers, subsequent to autoclaving at 120 °C for 20 min. After autoclaving, the grape must was analyzed chemically for sugar (°B), pH, alcohol (% *v*/*v*) and total acidity (g·L^−1^) using a density meter (Density meter DMA 35, Anton Paar, Graz, Austria), pH meter (Metrohm pH meter 632, Herisau, Switzerland), alcolyzer (Anton Paar, Graz, Austria) and minititrator (Hanna instruments minititrator HI 84502, Johannesburg, South Africa); respectively.

### 2.2. Inoculum Preparation

Cryopreserved non-*Saccharomyces* yeast (*n* = 5) (Table 1) and acetic acid bacteria (*n* = 5) (Table 2) cultures were used for this study. The yeast and bacteria cultures were individually grown in 400 mL YPD (Merck, Modderfontein, South Africa) and in 400 mL GM broth (glucose 0.8%, mannitol 1.7%, peptone 0.3%, yeast extract 0.5%) (Merck, Modderfontein, South Africa), respectively. The yeast and bacteria inoculums were incubated at 28 °C for 48 and 72 h respectively, prior to inoculation. 

The non-*Saccharomyces* yeasts were selected based on a previous screening investigation. The yeast used in this study were selected due to high acid formation on calcium carbonate agar plates, desired aroma of the final fermented product, osmophilic characteristics and the final concentration of the alcohol produced.

Similarly, acetic acid bacteria were obtained from previous isolation procedures on various sources; namely, grape pomace, healthy grapes and Shiraz wine. The bacteria used for this study were selected based on their ethanol oxidation rate when inoculated in diluted grape juice or their sugar utilization abilities in autoclaved grape juice.

### 2.3. Sterilization of Corncobs and Oak Wood Chips

**Figure 1:**
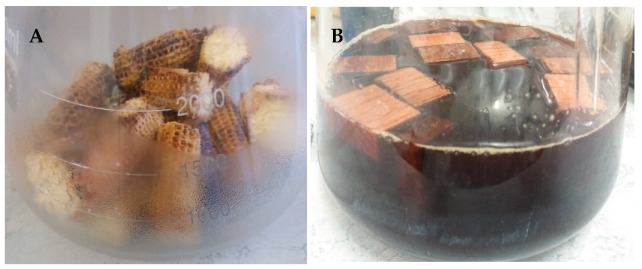
Corncobs and oak wood chips used in the study, (**A**) corncobs after autoclaving (**B**) oak wood chips in cooked grape must.

Corncobs (CC) were dried in the oven prior to cutting into smaller pieces using a cutting tool (sizes are shown in Table 3 and Figure 1). French oak wood chips (OWC) were used for this study. The OWC did not require any drying. Subsequently, the CC and OWC were separately transferred into 4 × 3 L Erlenmeyer flasks prior to autoclaving at 121 °C for 20 min. Autoclaving was repeated (*n* = 2) to maximize sterility. 

Yeast and bacteria were immobilized separately due to their difference in cell size. Furthermore, the differences in turbidity of the broth after yeast and bacteria cell growth was assumed to be a factor that might affect cell adsorption between the yeasts and bacteria. 

### 2.4. Cell Immobilization on Corncobs and Oak Wood Chips

After yeast and bacteria were fully-grown, the inoculums were mixed resulting in a 2 L consortium of yeast and bacteria (Table 1 and Table 2) (NB: yeast and bacteria consortiums were initially in separate flasks). Subsequently, 1 L of the yeast consortium was transferred into 3 L Erlenmeyer flasks, one containing CC and another one containing OWC. The same procedure was performed using the bacterial consortium. The yeast and bacterial cells were allowed to adsorb onto the surface of CC and OWC (serving as a solid support surface/bed) overnight at 28 °C. 

### 2.5. Quantification of Cells Adsorbed on Corncobs and Oak Wood Chips Prior- and Post-Fermentation

The number of cells adsorbed on the CC and OWC for individual yeasts and bacteria were quantified using the dry cell weight method adapted from Stone et al. [26] and Nguyen et al. [27], with minor modifications. Prior to this, the yeast and bacterial cell concentration (Table 3) in liquid suspension were individually quantified following the procedure described in Hutchinson et al. [28]. Furthermore, the yeast and bacteria were individually studied to assess the variations in cell adsorption capabilities. Yeast and bacteria were grown individually following the procedure described in Section 2.2. Subsequently, the yeast and bacteria cells were individually adsorbed onto the CC and OWC following the procedure described in Section 2.4. CC and OWC were removed from the broth and dried in an oven set at 40 °C. The adsorbents were weighed daily until a stationery weight was reached. 

To assess the quantity of cells adsorbed post fermentation, the CC and OWC were transferred into grape must and allowed to ferment for 20 days. Subsequently, the adsorbents were extracted and dried at 40 °C until a stationery weight was reached. To determine the number of cells adsorbed for the procedures, the difference of the weight of the adsorbents prior and post adsorption was computed. 

### 2.6. Inoculation

After the adsorption process, the OWC and CC were extracted from the broth and allowed to dry for 4 h. A 3 L fermentation volume was used for this study. Four pieces of corncobs (2 pieces yeast, 2 pieces Acetic Acid Bacteria) (Table 2) were used for inoculation in one flask. Twenty pieces of oak wood chips (Figure 1B) were used for inoculation in another flask (10 pieces yeast, 10 pieces AAB) (Table 2).

#### 2.6.1. Phase 1: Static vs. Agitated Fermentations

Static and agitated fermentations were both incubated at 28 °C with agitated fermentations at 135 rpm. Agitated fermentations were conducted using an orbital shaker (FMH 200, FMH Instruments, Cape Town, South Africa). Under both static and agitated conditions, CC, OWC and FFC fermentations were conducted in triplicate. 

#### 2.6.2. Phase 2: Effect of Aeration

Air pumps (Resun^®^ AC 9906, Longgang, Shenzhen, China) were used to sparge air into the Erlenmeyer flasks at different airflow rates, i.e., low aeration (LA = 0.15 vvm min^−1^) and high aeration (HA = 0.30 vvm min^−1^). Erlenmeyer flasks were covered with loose cotton wool stoppers and fermentations were conducted in triplicate.

### 2.7. Data Handling 

Data was analyzed and computed using Microsoft Excel v2016 (Microsoft, Redmond, Washington, DC, USA). Substrate (*S*) (sugar) consumption rates and product (*P*) (acetic acid) formation rates were calculated using Equations (1) and (2) respectively, with an assumption that the initial reaction rate determines the overall fermentation rate. NB: change in time (*t*) was expressed as *dt* for both equations. Furthermore, relative differences as employed in other studies [28,29] were calculated using Equations (3) and (4) in order to show the significance of the differences observed under the different conditions studied. All results were the average of three biological repeats accounting for standard deviations which were calculated using Microsoft Excel v2016.

(1) $$r_{s}=\frac{dS}{dt}$$

(2) $$r_{p}=\frac{dP}{dt}$$

(3) Absolute difference=Amount of increase−Reference amount

(4) $$\mathrm{Relative}\;\mathrm{difference}=\frac{\mathrm{Absolute}\;\mathrm{difference}}{\mathrm{Reference}\;\mathrm{amount}}\;\times \;100$$

## 3. Results and Discussions

### 3.1. Phase 1: Non-Aerated Fermentations

#### 3.1.1. Static vs. Agitated Fermentations

TBV fermentation is normally carried-out under static conditions using batteria. This process is slow and inexpensive [1,9]; however, a rapid fermentation period for TBV is not a fundamental objective, since TBV is matured for a minimum of 12 years after fermentation [1], while BSV can be sold without ageing. 

**Figure 2:**
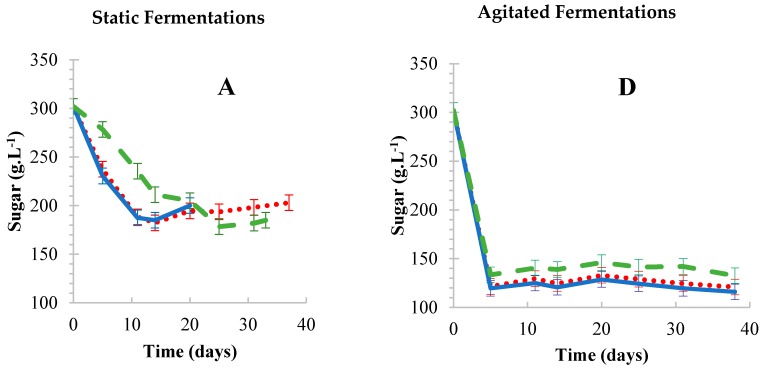

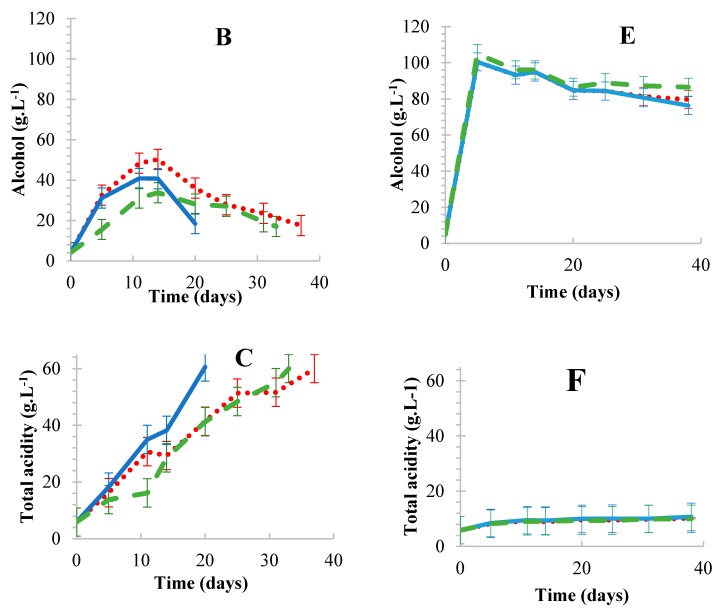
Chemical developments under static (**A**–**C**) and agitated (**D**–**F**) conditions. Sugar (**A**,**D**), alcohol (**B**,**E**) and total acid (**C**,**F**) developments during fermentation. 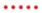 Oak wood chips, 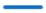 Corncobs, 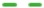 Free-floating cells. Results are the average of three biological repeats accounting for standard deviation.

Rapid vinegar production is a major goal for industrial processes. BSV can also be produced rapidly if the conditions are ideal for the microorganisms used. For the purposes of this study, it was important to evaluate the effects that agitation could have on BSV production in contrast to stationary fermentations. Agitation is also recommended for spirit industrial vinegar production to generally shorten the fermentation period [30]. In the current study, the sugar consumption rates for all treatments under agitated conditions ranged between 6.36 and 7.12 g·L^−1^·day^−1^ whereas 2.68 and 6.10 g·L^−1^·day^−1^ were observed under static conditions. The relative differences for sugar consumption between FFC and immobilized cells were calculated to be 14% (OWC and FFC) and 41% (CC and FFC) under static conditions, while the relative differences under agitated conditions were calculated to be 5% (OWC and FFC) and 9% (CC and FFC) under static conditions. The ethanol production step of the process was more effective under agitated conditions, as compared to static conditions (Figure 2A,D). However, ethanol formation/consumption rates were not calculated, since alcohol is an intermediate product/substrate which is produced and consumed simultaneously. Furthermore, acetic acid production was unsatisfactory for all treatments under agitated conditions (Figure 2F). Agitation resulted in acetification rates ranging from 0.11 to 0.13 g·L^−1^·day^−1^, with the highest acetic acid concentration of 10.7 g·L^−1^ being achieved at day 35. However, for a successful BSV fermentation, an acetic acid concentration of at least 50 g·L^−1^ is usually required [10]. Hutchinson et al. [17] made similar observations when using cell immobilization with calcium alginate beads for BSV production. It is unclear if these observations were species (acetic acid bacteria) dependent or due to the agitation settings used. In light of these observations, other vinegar studies have successfully applied agitation speeds between 100 [31] and 200 rpm [32], with industrial spirit vinegars successfully employing maximum agitation up to 900 rpm [30,33,34]. This suggested that the current agitation setting could not have negatively impacted the microbial activity in the fermentations. Furthermore, most vinegar production systems generally employ agitation and aeration simultaneously [34,35]; therefore, it is important to consider that agitation might only be beneficial under such settings. 

Figure 2C shows successful acetification/alcohol oxidation profiles for all treatments under static fermentations. Total acid formation rates ranged from 1.46 and 2.70 g·L^−1^·day^−1^ for all of the treatments studied. The relative differences for total acid formation between agitated and static fermentations were 93%, 92% and 95% for FFC, OWC and CC, respectively. Furthermore, the low substrate (sugar) consumption rates (2.68–6.10 g·L^−1^·day^−1^) resulted in much lower alcohol formation rates (Figure 2B) but high total acid formation rates (Figure 2C). These observations could mean that the AAB microbial activity was ideal at low alcohol concentrations, suggesting that the agitation speed should be optimized with the aim of reducing alcohol formation rates, in order to benefit the AAB used. Furthermore, if agitation is not suitable for BSV fermentations, regardless of the speed, this might benefit producers, because agitation is energy intensive and the cost input might escalate, not only due to the energy usage but also due to the mechanical equipment required for agitation. 

#### 3.1.2. Effect of the Adsorbents Used

The adsorption technique for cell immobilization has been studied using several inert materials for various vinegar production systems [31,32,36,37]. In the current study, CC were compared to OWC for cell affinity and influence on BSV production. These materials differ in terms of their biological composition as they have different physical and structural attributes, as well as adsorption capacity and surface chemistry. 

Since agitated fermentations did not meet the required performance for BSV production, i.e., achieving 60 g·L^−1^ acetic acid, a more comprehensive discussion is provided in this section on the fermentations that were only conducted under static conditions. Additional reasons attributed to the unsuccessful agitated fermentations, which were characterized by low acetification rates (0.11 to 0.13 g·L^−1^·day^−1^) may be due to the interference of cell adsorption by agitation conditions, which eventually resulted in freely suspended cells. 

Moderately higher sugar consumption rates of 8.14 and 5.14 g·L^−1^·day^−1^ for OWC and CC, respectively, were observed at the initial stages (day 0–14) of the fermentation process (Figure 2A). Alcohol formation rates were also moderately higher for CC and OWC fermentation than FFC fermentations (Figure 2B). While, total acid formation for cells immobilized on CC was consistently higher throughout the fermentation process, with total acid formation rates of 2.7 g·L^−1^·day^−1^ being achieved in the shortest fermentation period (20 days). Comparatively, FFC and OWC fermentations resulted in lower total acid formation rates of 1.64 and 1.46 g·L^−1^·day^−1^ with a longer fermentation period of 33 and 37 days, respectively. Furthermore, the relative differences between FFC and immobilized cells were 11% (OWC and FFC) and 39% (CC and FFC). Therefore, it was deduced that cells adsorbed on CC fermentations led to the highest microbial activity because of the adsorbents’ attributes, i.e., rough surface and porosity, which improved the adsorption of cells. As a result, the microbial consortium was able to form a highly effective community when adsorbed onto the CC. Contrarily, the OWC are relatively smoother than the porous CC, which consequently led to the poor adsorption of cells. 

Cell immobilization on CC was also studied for the production of tea vinegar, with acetification rates of 2.88 g·L^−1^·day^−1^ being obtained [38]. Several studies often use wood shavings for cell immobilization instead of OWC. For example, Thiripurasundari and Usharani [39] and Kocher and Dhillon [40] reported acetification rates of 0.24 g·L^−1^·day^−1^ and 5.76–22.8 g·L^−1^·day^−1^ when using wood shavings for the production of cashew apple vinegar and sugar cane vinegar, respectively. It is not clear as to the reasons why the acetification rates for these two studies were different; however, other factors such as substrate availability, bacteria strain used and other fermentor conditions might have played a role. Furthermore, wood shavings and OWC may not be comparable materials due to their differentiated physical properties, and thus, differentiated adsorption capabilities. 

Table 4 shows a comparison of other studies which investigated the effect of cell immobilization by adsorption in other vinegar production systems. The current work established a direct relationship between cell immobilization and higher acetification rates. Similarly, other studies [41] also reported a proportional relationship between cell immobilization and acetification rates, with the fibrous bed being reported to show a conspicuous increase in acetification compared to FFC (Table 4). Furthermore, when a loofa sponge was used, it led to increased acetification rates [42] which were similar to the rates observed in the current study at static conditions. 

Table 4 also shows that agitation speeds of 100 rpm, including reciprocating shaking speed of 60 rpm were employed effectively in other studies. These findings illustrate that agitation may also be dependent on several other factors, such as the microorganisms used, the type of vinegar being produced and other fermentor conditions.

### 3.2. Phase 2: Aerated Fermentations

#### 3.2.1. Effect of Aeration Rates

**Figure 3:**
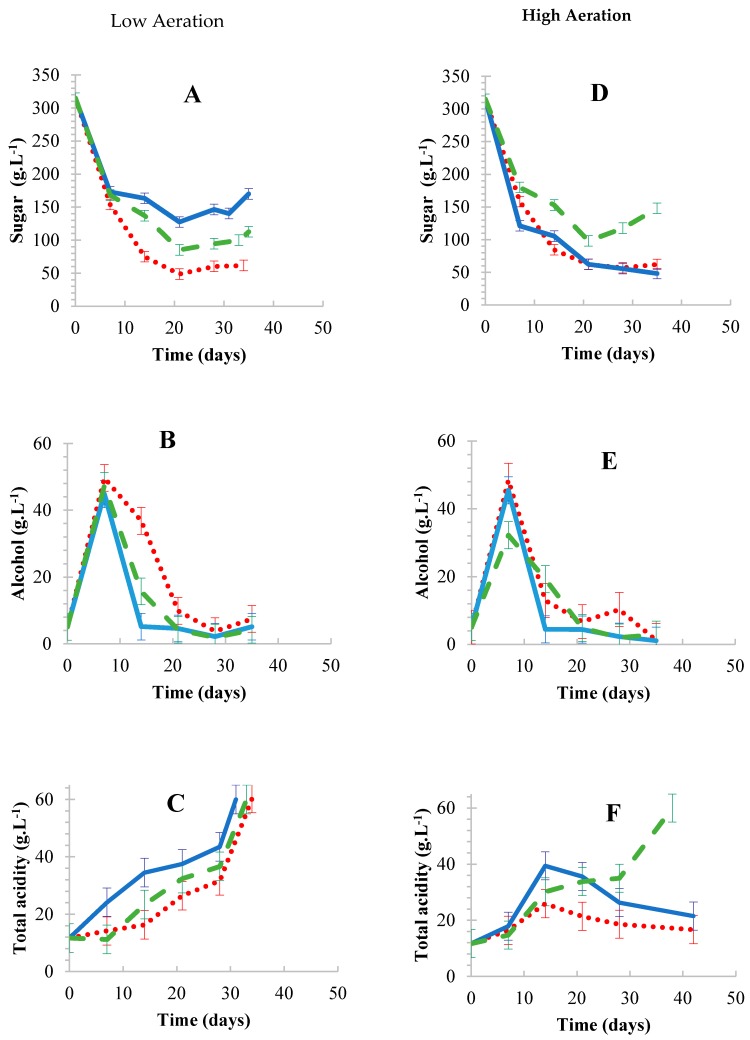
Chemical developments under low (**A**–**C**) and high (**D**–**F**) aeration. Sugar (**A**,**D**), alcohol (**B**,**E**) and total acid (**C**,**F**) developments during fermentation. 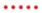 Oak wood chips, 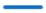 Corncobs, 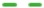 Free-floating cells. Results are the average of three biological repeats ± standard deviation.

Aeration is generally a fundamental aspect in most vinegar fermentation systems [43,44]. Aeration systems vary between transferring pure oxygen or air, with pure oxygen systems being noted as expensive due to the cost of technical grade oxygen [45] and deleterious effects such as hyperoxia which leads to cellular death. For this study, aeration with air only was investigated as it was deemed cost effective. It was observed that both high aeration (HA) and low aeration (LA) led to similar alcohol formation/consumption profiles (Figure 3B,E). The observations were surprising since alcoholic fermentation is often performed under anaerobic conditions or low dissolved oxygen conditions [46,47]. Consequently, yeast performance was expected to also be negatively affected by HA, albeit that yeasts are generally characterized as facultative anaerobes and some yeasts can still survive or grow under aerobic conditions [48,49,50,51]. Since only air (with 21% oxygen) was used, it was also plausible that different alcohol consumption profiles could have been observed if technical grade oxygen was tested—a condition that can exacerbate hyperoxia. 

Nevertheless, total acid formation, which is a key step in BSV production was higher (1.42–1.56 g·L^−1^·day^−1^) at LA (Figure 3C) as compared to HA (0.14–1.2 g·L^−1^·day^−1^) (Figure 3F) for cell immobilized treatments. However, FFC completed the fermentations under both aeration settings with acetification rates of 1.27 and 1.46 g·L^−1^·day^−1^ for HA and LA, respectively. Additionally, over oxidation has been reported as a common challenge in most vinegar production systems [10]. It seems unlikely that the excessive/increased oxidation rates may have affected the fermentations in this study; since the alcohol profiles under HA and LA conditions (Figure 3B,E) were similar to some degree. Other studies reported higher acetification rates compared to the current study when aeration was employed. Rubio-Fernández et al. [44] reported acetification rates of 17.24 and 32.4 g·L^−1^·day^−1^ for wine vinegar production when air and oxygen rich air (0.06 vvm) were used respectively. Additionally, Qi et al. [52] reported acetification rates of 43.44 g·L^−1^·day^−1^ for industrial vinegar production when oxygen (0.13 vvm m^−1^) was used. It is important to mention that the observations between the current study and other studies are influenced by several factors, which include media composition and the AAB strains used. The most obvious influential factor is that BSV fermentation involves high sugar concentration/high osmotic pressures with the simultaneous involvement of non-*Saccharomyces* yeasts and AAB. Furthermore, spirit vinegar production is generally faster than the production of other vinegars. It is unfortunate that there are no similar BSV studies with which to compare the current data, due to the traditional techniques used for Balsamic vinegar production. 

#### 3.2.2. Performance of Adsorbents Used Under Aerated Conditions

When cells adsorbed on the different materials were evaluated at the initial stages of fermentation, sugar consumption showed similar profiles with sugar consumption rates between 20.15 to 27.2 g·L^−1^.day^−1^ under both aeration (HA and LA) settings for all treatments (Figure 3A,D). After 7 days, sugar consumption at LA was higher for OWC fermentations with rates between 3.32 g·L^−1^·day^−1^ followed by FFC and CC with a sugar consumption rate of 1.80 and 0.11 g·L^−1^·day^−1^ respectively (Figure 3A). On the other hand, HA, CC and OWC fermentations led to relatively similar results, as well as the highest sugar consumption rates (2.70–3.46 g·L^−1^·day^−1^) compared to FFC (1.28 g·L^−1^·day^−1^) (Figure 3D). The lowest sugar consumption rates were observed in FFC fermentations under HA with trends that we cannot compare with any previously available literature. However, it is possible that the activity of freely suspended yeast cells under HA was reduced by AAB FFC that eventually led to a higher production of acetic acid. Overall, sugar consumption rates were 4.14, 5.78 and 7.4 g·L^−1^·day^−1^ under LA and 7.62, 4.82 and 7.23 under HA for CC, FFC and OWC, respectively. The relative differences for sugar consumption between LA and HA fermentations were 2%, 17% and 46% for OWC, FFC and CC; respectively. Furthermore, alcoholic fermentation was successful on all immobilized and FFC fermentations for both aeration settings. Alcohol formation/consumption profiles were moderately similar for all treatments studied under both aeration settings. 

Total acid formation provided an insight on the productivity of the process evaluated. It was evident, that CC fermentations had the highest total acid formation at LA, followed by FFC and then OWC fermentations. The differences observed were minor, since the total formation rates were 1.56, 1.46 and 1.42 g·L^−1^·day^−1^ with a fermentation period of 31, 33 and 34 days for CC, FFC and OWC fermentations, respectively. Under HA, only FFC fermentations completed the fermentation with an acetification rate of 1.2 g·L·day^−1^ in 38 days, while CC and OWC fermentations initiated by showing an increase in total acid subsequent to its decrease from day 14 to 42. It was not well understood as to how the FFC fermentations were able to reach the required levels of total acid concentration. A credible assumption could be that HA may have disrupted the adsorbed cells, thus causing and maintaining a low microbial activity. Overall, based on the results obtained, it was evident that agitation and HA did not favor the production of BSV, where cell immobilization by adsorption was used.

### 3.3. Variations in Cell Adsorption Capabilities among the Yeast/Bacterial Species Used

#### 3.3.1. Individual Yeast Adsorption on Corncobs and Oak Wood Chips

The phenomenon of adsorption efficiency is critical to the current study. The adsorption by different microorganisms varied due to cell affinity differences to the adsorbents surface used. However, it is possible that microorganisms employed will adsorb differently when tested as a consortium. With regards to yeast adsorption on CC, it was observed that *C. zemplinina* had the lowest number of cells adsorbed prior and post fermentation. *Z. bailii* initially exhibited low cell adsorption before fermentation; however, a relatively higher cell adsorption (92.93%) after fermentation was observed. Yeasts species such as *C. pulcherrima, H. guilliermondii* and *K. apiculata* showed a noticeably high cell adsorption prior and post fermentation (Table 5). 

Furthermore, yeast cell adsorption was different for both OWC and CC. Some yeast species such as *C. zemplinina* (59.54%) and *Z. bailii* (28%) showed a relative decrease in cell adsorption post fermentation (Table 5). The trends showed that *C. zemplinina* and *Z. bailii* might have low cell affinity to the smooth ‘easy-to wash-off’ OWC surface during the fermentation compared to CC surfaces. On the other hand, *C. pulcherrima* (87.78%)*, H. guilliermondii* (3.00%) and *K. apiculata* (48.67%) showed a relative increase in cell adsorption capacity during the fermentation, with *H. guilliermondii* showing the lowest adsorption. Overall, the cells adsorbed (g·g^−1^) on CC and OWC were not comparable since they have different densities, including surface chemistry properties. 

#### 3.3.2. Individual Bacteria Adsorption on Corncobs and Oak Wood Chips

The adsorption of bacteria onto CC and OWC was also evaluated (Table 6). *A. pasteurianus* and *A. malorum* appeared to have the highest (0.1461 and 0.095 g·g^−1^, respectively) cell adsorption efficiency on CC before fermentation (Table 6). *K. baliensis, G. cerinus* and *G. oxydans* had relatively similar adsorption efficiency on CC before fermentation, with relatively low variations (97.76%, 99.26%, 96.12%, respectively) observed post fermentation. Similar decreases in adsorption efficiency patterns on OWC were observed between *A. malorum* and *K. baliensis* (Table 6). *A. pasteurianus* had the lowest initial cell adsorption (0.0020 g·g^−1^) before fermentation; however, it had the highest percentage cell increase (98.87%) after fermentation. *G. cerinus* and *G. oxydans* showed a similar pattern before and after fermentation, with 52.40% and 56.33% in cell adsorption after fermentation. Other studies have investigated the cell adsorption on CC and wood shavings; however, these studies did not report the number of cells adsorbed on the adsorbents used, which leads to the lack of comparative data. 

Overall, these results showed a successful adsorption on CC and OWC for both yeasts and bacteria, but with varying adsorption efficiency. However, the data also displayed an unstable cell adsorption profile on OWC, since a decrease in cell adsorption was observed for some yeast and bacterial species. The data further suggested a more sustainable approach on the reusability of immobilized cells on CC and OWC treatments.

## 4. Conclusions

According to all the results obtained, it is evident that cell immobilization improves acetification rates during BSV fermentation. However, the highest acetification rates were obtained under static and non-aerated conditions. Corncobs were observed to be the most suitable material for cell immobilization presumably due to their physical structure. Consequently, the shortest fermentation period was 20 days when cells were immobilized on corncobs under static fermentation conditions. A study to investigate the concurrent optimization of agitation and aeration as well as the effect of corncobs and oak wood chips on organoleptic properties is recommended. The current study therefore, serves as a foundation for cell immobilization by adsorption on materials during balsamic-styled vinegar production.

## Figures and Tables

**Table 1:** Non-*Saccharomyces* yeast used in the study.

Non-*Saccharomyces* Yeast		
Identity	ARC Accession Numbers	Origin
*Candida pulcherrima*	Y0839	Chardonnay grapes
*Candida zemplinina*	Y1020	Chardonnay grapes
*Hanseniaspora guilliermondii*	Y0558	Cabernet Sauvignon grapes
*Kloeckera apiculata*	C48V19	Chardonnay grapes
*Zygosaccharomyces bailii*	C45V69	Chardonnay grapes

ARC: Agricultural Research Council of South Africa.

**Table 2:** Acetic acid bacteria used in the study.

Acetic Acid Bacteria			
Identity	ARC Accession Numbers	NCBI Accession Numbers	Origin
*Acetobacter pasteurianus*	171/19	CP 021922.1	Healthy grapes
*Acetobacter malorum*	172/36	KU 686765.1	Shiraz wine
*Kozakia baliensis*	179/48	CP 014681.1	Grape pomace
*Gluconobacter cerinus*	126/34	KX 578017.1	Shiraz wine
*Gluconobacter oxydans*	172/36	LN 884063.1	Grape pomace

NCBI: National Center for Biotechnology Information.

**Table 3:** Size of corncobs and oak wood chips used in the study.

Adsorbents	Length (cm)	Width/Diameter (cm)	Circumference/Perimeter (cm)	Surface Area of One Piece (cm^2^)	Surface Area of all Adsorbents Used (cm^2^)
Corncobs	6.00 ± 1.05	4.00 ± 0.66	12.57	100.53	402 (4 pieces)
Oak woodchips	2.90 ± 0.65	1.80 ± 0.53	9.60	19.64	392 (20 chips)

Length and width results are the average of repeats ± standard deviations.

**Table 4:** A comparison of the performance of immobilized cells in other studies.

Product	Adsorbent	Agitation	Acetification Rate (g·L^−1^·day^−1^)	Reference
Balsamic-styled vinegar	Corncobs Oak wood chips	135 rpm	FFC: 0.11 CC: 0.13 OWC: 0.11	Current study
Acetic acid from lactose and milk permeate	Fibrous-Bed/matrix	100 rpm	FFC (lactose): 1.44 FFC (milk permeate): 1.92 IC (Lactose): 12.96 IC (milk permeate) 7.2	[31]
Rice wine vinegar	Loofa sponge	1 Hz reciprocating shaking rate = 60 rpm	IC: 1.68–2.4	[42]

IC: immobilized cells; CC: Corncobs; FFC: free-floating cell; OWC: oak wood chips.

**Table 5:** Evaluation of yeast cells adsorbed on corncobs and oak wood chips.

		Yeast Cells Adsorbed on Corncobs			Yeast Cells Adsorbed on Oak Wood Chips		
Identity	Cell Concentration YPD Broth (Cells·mL^−1^)	Before Fermentation (g·g^−1^)	After Fermentation (g·g^−1^)	Relative Difference (%)	Before Fermentation (g·g^−1^)	After Fermentation (g·g^−1^)	Relative Difference (%)
*Candida pulcherrima*	2.34 × 10^5^	0.0371	1.1580	96.80	0.0214	0.1751	87.78
*Candida zemplinina*	7.40 × 10^5^	0.0006	0.0290	97.93	0.0903	0.0566	−37.32
*Hanseniaspora guilliermondii*	7.70 × 10^5^	0.0110	0.7906	98.61	0.1001	0.1032	3.00
*Kloeckera apiculata*	1.95 × 10^6^	0.0283	1.6955	98.33	0.0926	0.1804	48.67
*Zygosaccharomyces bailii*	3.97 × 10^5^	0.0400	0.5661	92.93	0.1785	0.1393	−21.96

**Table 6:** Evaluation of bacterial cells adsorbed on corncobs and oak wood chips.

		Bacteria Cells Adsorbed on Corncobs			Bacteria Cells Adsorbed on Oak Wood Chips		
Identity	Cell Concentration GM Broth (Cells·mL^−1^)	Before Fermentation (g·g^−1^)	After Fermentation (g·g^−1^)	Relative Difference (%)	Before Fermentation (g·g^−1^)	After Fermentation (g·g^−1^)	Relative Difference (%)
*Acetobacter pasteurianus*	3.97 × 10^5^	0.1461	1.1968	87.79	0.0020	0.1772	98.87
*Acetobacter malorum*	1.25 × 10^6^	0.0948	1.0882	91.29	0.1851	0.1545	−16.53
*Kozakia baliensis*	3.13 × 10^5^	0.0260	1.1589	97.76	0.1224	0.1122	−8.33
*Gluconobacter cerinus*	2.22 × 10^6^	0.0116	1.5618	99.26	0.1498	0.3147	52.40
*Gluconobacter oxydans*	6.38 × 10^5^	0.0426	1.0977	96.12	0.1008	0.2308	56.33
